# Synthesis of Boron Nitride Nanotubes Using Plasma-Assisted CVD Catalyzed by Cu Nanoparticles and Oxygen

**DOI:** 10.3390/nano11030651

**Published:** 2021-03-08

**Authors:** Tatsuya Shiratori, Ichiro Yamane, Shoto Nodo, Ryo Ota, Takashi Yanase, Taro Nagahama, Yasunori Yamamoto, Toshihiro Shimada

**Affiliations:** 1 Graduate School of Chemical Science and Engineering, Hokkaido University, Kita 13 Nishi 8, Sapporo 060-8628, Japan; takoyaki.oct@gmail.com (T.S.); 16yamane@gmail.com (I.Y.); punkan-369@eis.hokudai.ac.jp (S.N.); yanase42@eng.hokudai.ac.jp (T.Y.); nagahama@eng.hokudai.ac.jp (T.N.); yasuyama@eng.hokudai.ac.jp (Y.Y.); 2 Center for Advanced Research of Energy and Materials, Faculty of Engineering, Hokkaido University, Kita 13 Nishi 8, Sapporo 060-8628, Japan; oota@eng.hokudai.ac.jp; 3 Division of Applied Chemistry, Faculty of Engineering, Hokkaido University, Kita 13 Nishi 8, Sapporo 060-8628, Japan

**Keywords:** boron nitride nanotube, plasma chemical vapor deposition (CVD), borazine, Cu, Cu_2_O, core-shell structure

## Abstract

We found that oxidized Cu nanoparticles can catalyze the growth of boron nitride nanotubes from borazine via plasma-assisted chemical vapor deposition. The Raman spectra suggest that the formation of thin-walled nanotubes show a radial breathing mode vibration. The presence of oxygen in the plasma environment was necessary for the growth of the nanotubes, and a part of the nanotubes had a core shell structure with a cupper species inside it. In atomic resolution transmission electron microscope (TEM) images, Cu_2_O was found at the interface between the Cu-core and turbostratic BN-shell. The growth mechanism seemed different from that of carbon nanotube core-shell structures. Therefore, we pointed out the important role of the dynamic morphological change in the Cu_2_O-Cu system.

## 1. Introduction

Boron nitride nanotubes (BNNTs) [1,2,3] have structures similar to carbon nanotubes (CNTs) with the hexagonal boron nitride (h-BN) sheet rolled up to make cylinders. The BNNTs have a large bandgap (5–6 eV) and exhibit good electrical insulation irrespective of their chirality [4]. They also show a high thermal conductivity, high mechanical strength [5], and chemical stability [6]. BNNTs with a specific chirality exhibit a piezoelectricity [7]. It also adsorbs hydrogen molecules [8]. Therefore, it is expected that BNNTs have applications in various fields including thermal radiators, insulators, sensors, energy harvesting devices, hydrogen storage, and bio-related devices.

The synthesis of BNNTs has been attempted using various approaches such as arc discharge [9], laser ablation [10,11,12], ball milling followed by annealing [13], chemical vapor deposition (CVD) with catalysts [14,15,16,17,18,19,20,21], CVD on carbon nanotubes [22], and inductively coupled plasma [23]. They can be classified into seral categories from a solid boron (B) source or from a molecular B source, and with or without a catalyst. The solid B source (boron or h-BN) requires very high temperatures. The synthesis with the molecular B source often involves B_2_H_6_ and NH_3_, which are toxic and explosive. Another molecular source, BH_3_NH_3_, forms liquid droplets when vaporized, which forms h-BN nanoparticles as an impurity of the BNNTs. Borazine (B_3_N_3_H_6_) causes less problems in this sense [24]. The formation of nanoscale seeds seems essential for the formation of BNNTs and they are sometimes boron nanoparticles and sometimes other nanoparticle catalysts. The search for the BNNT catalyst is still underway; Ni [24], Fe [25,26], Fe_2_O_3_ [27], and Mg [28] were studied, and, recently, Cu nanoparticles joined them [22] because Cu foil is one of the best catalysts and substrates for h-BN mono-layer synthesis [29,30].

In this paper, we report the plasma-assisted chemical vapor deposition (CVD) using borazine as the boron and nitrogen source and Cu as the catalyst. We realized the formation of a Cu-core and BNNT shell structure by adding oxygen gas to the plasma. The process temperature is low compared to the previous reports, and the growth mechanism of this kind has not been reported to the authors’ knowledge.

## 2. Materials and Methods

The advantage of borazine is that it is a stable volatile liquid and can be supplied to the reaction system in a stoichiometric and reproducible manner. Borazine is not highly toxic nor explosive, and it can be easily prepared from common chemicals [31]. Borazine was synthesized by heating (NH_4_) _2_SO_4_, NaBH_4_, and tetraglyme (purchased from FUJIFILM Wako Pure Chemical, Osaka, Japan), with stirring at 135 °C and a controlled temperature profile. It was distilled using sequential cold traps. The purity was confirmed using nuclear magnetic resonance (NMR). The product was sealed under an inert gas and stored in a low temperature freezer before use. It was handled in an Ar glove box and was placed in a glass tube welded to an ultrahigh vacuum flange connected to a metal bellows valve.

**Figure 1:**
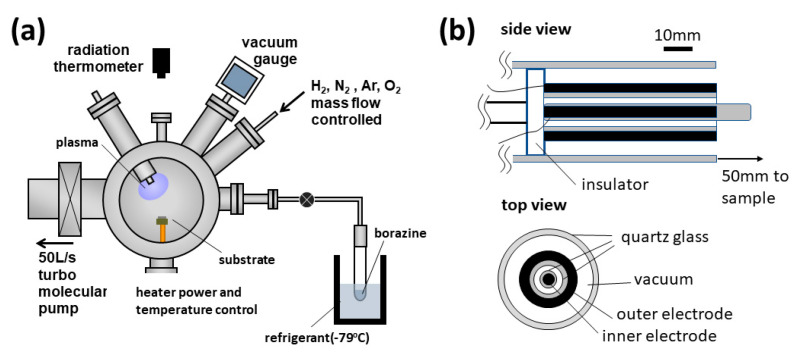
Schematic of (**a**) the reaction chamber and (**b**) the dielectric barrier discharge (DBD) source. The voltage was applied between outer and inner electrodes.

We used a vacuum chamber as shown in Figure 1a for the BNNT synthesis. Borazine was put in a glass tube welded to an ICF-34 flange with an ultrahigh vacuum (UHV) compatible bellows valve in an Ar glove box. This borazine source was connected to the chamber. Since the vapor pressure of borazine is rather high at room temperature, the borazine glass tube was cooled to lower than −60 °C). We prepared the borazine-containing plasma by home-made dielectric barrier discharge (DBD) source (Figure 1b) operated at a voltage of 700–950 V with alternating polarity at 50 Hz.

We used CuO nanoparticles (20-nm diameter, purchased from Sigma-Aldrich Japan, Tokyo, Japan) as the catalyst for the growth. They were coated on a Si wafer (10 mm × 5 mm × 0.5 mm) by immersing it in a water suspension of the CuO nanoparticles in an ultrasonic bath. It was introduced to a vacuum chamber for the plasma CVD and pumped by a turbo molecular pump. Three types of CuO_x_ nanoparticles were used as the catalysts. The first one was CuO, which was used as received. The second one was Cu, which was prepared by heating to 500 °C and processed by H_2_ plasma at a pressure of 15 Pa for 30 min to reduce the CuO to Cu. The third was Cu_2_O formed on Cu nanoparticles, which were formed by adding O_2_ gas in the plasma environment. During the CVD, the substrate was heated to 600 °C in a vacuum, then the plasma CVD started in the mixture of N_2_ (or Ar) + O_2_ + borazine at a total pressure of 15–45 Pa.

We used X-ray diffraction (XRD, Rigaku Miniflex, Cu Ka, Tokyo, Japan), TEM (JEOL JFM-2010, 200 keV, Tokyo, Japan), STEM (FEI TITAN 3 G2 60-300, Hillsboro, OR, US) operated at 60 keV to reduce the electron beam damage), and Raman microscopy (Renishaw Invia, operated with 532 nm laser, Wotton-under-Edge, Gloucestershire, UK) to characterize the obtained materials.

## 3. Results and Discussion

**Figure 2:**
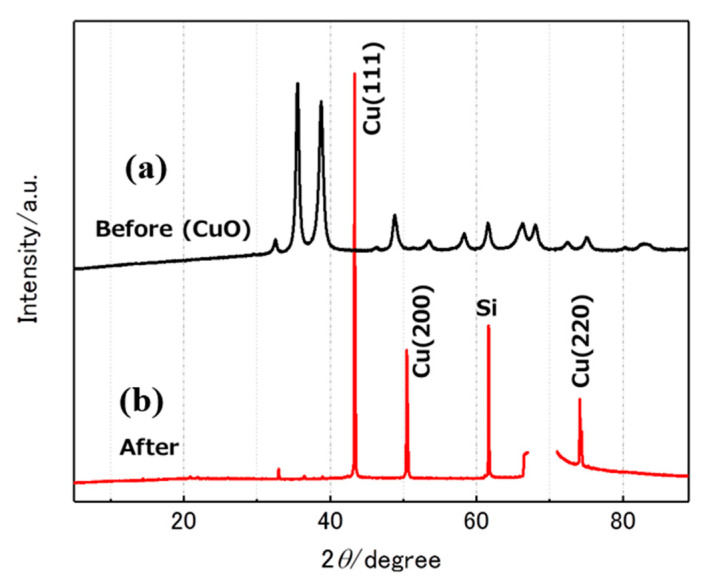
X-ray diffraction (XRD) pattern of CuO nanoparticles (**a**) before and (**b**) after H_2_ plasma treatment.

We first tried the CVD with untreated CuO nanoparticles. We found that amorphous BN was formed on the Cu nanoparticles, but no wire-like structures were formed. We next tried to use Cu nanoparticles. Prior to the CVD, we confirmed the effect of the hydrogen plasma treatment to reduce the CuO nanoparticles to Cu. Figure 2 shows the XRD pattern before and after the treatment. Diffraction peaks corresponding to CuO in Figure 2a (before the treatment) disappeared in Figure 2b (after the treatment), and peaks corresponding to Cu appeared. 

This result clearly indicated that CuO nanoparticles were reduced to Cu. This is due to the elevated temperature and reducing potential of hydrogen radicals. We used these Cu particles for the CVD. Without this hydrogen plasma treatment, no BNNTs were obtained, even when using the same procedure as described in the following sections.

**Figure 3:**
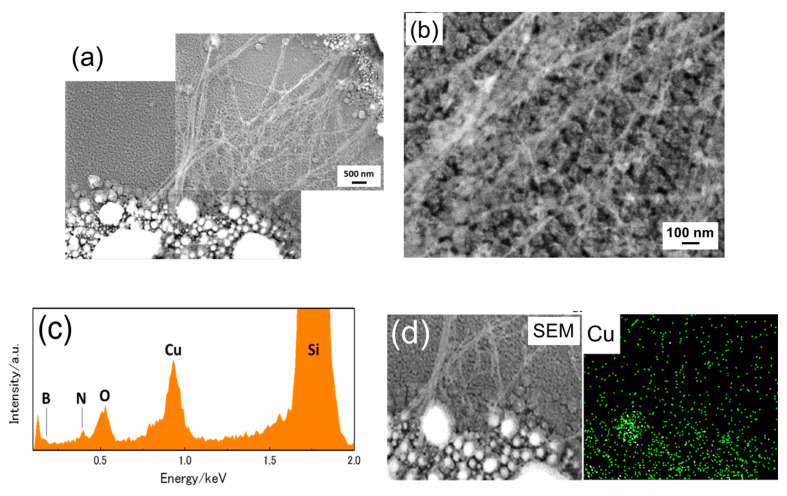
SEM images of the as-grown boron nitride nanotubes (BNNTs) (N_2_:O_2_:borazine = 3:1:1) at (**a**) low and (**b**) high magnification. (**c**) energy dispersion specroscopy (EDS) spectrum and (**d**) EDS map.

Figure 3a,b shows SEM images after the plasma CVD with N_2_ + O_2_ + borazine (9 Pa:3 Pa:3 Pa = 3:3:1)) under the optimized conditions. Web-like wires were observed on the surface. The wires appeared to connect two round-shaped objects. The wires or their bundle had a diameter of single to several 10s of nm. Figure 3c shows the energy dispersion specrum (EDS) spectrum of the web-like structural region. We detected the existence of boron and nitrogen. The EDS mapping image around the round objects is shown in Figure 3d. It is clearly seen that the round objects were made of Cu, suggesting that the BNNT was synthesized by Cu catalysts. However, it was difficult to further examine the thin wires using microscopy, because they could not be transferred to TEM grids.

**Figure 4:**
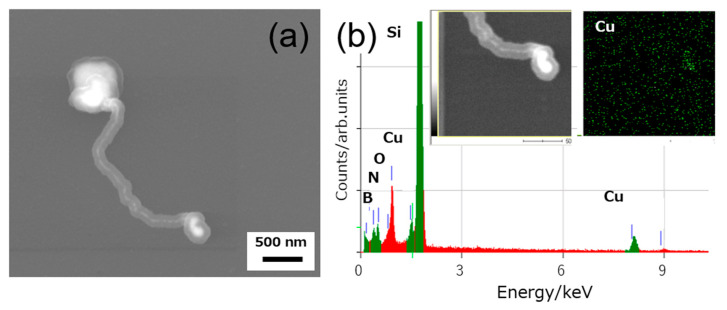
SEM image (**a**) and EDS spectrum and mapping (**b**) of material with Ar:O_2_:borazine = 5:3:17.

Figure 4a shows an field emission (FE)-SEM image of another nanostructure prepared under slightly different conditions (Ar:O_2_:borazine = 5 Pa:3 Pa:17 Pa, 600 °C). The structure had a greater size compared to the thin wires in Figure 3. It shows an irregular tubular shape and has two swollen structures at its ends. Two distinct contrasts are noted in the core-shell structure; one is gray and the other is white. An EDS analysis shown in Figure 4b and mapping of the Cu distribution (inset) indicated that the gray shell and white core are made of BN and Cu, respectively. We found that without oxygen, these wire-like nanostructures were not formed.

The process window for obtaining thin nanowires in Figure 3 was narrow. It seems that the room temperature, among other parameters, affects the parameter through the local vapor pressure of borazine and subtle change of the substrate temperature. However, thick core-shell wires are formed with a wide range of the partial pressures such as N_2_:O_2_:borazine = 5 Pa:13 Pa:17 Pa, 25 Pa: 5 Pa:10 Pa, or 20 Pa:10 Pa:10 Pa. N_2_ can be replaced to Ar for thick wires.

**Figure 5:**
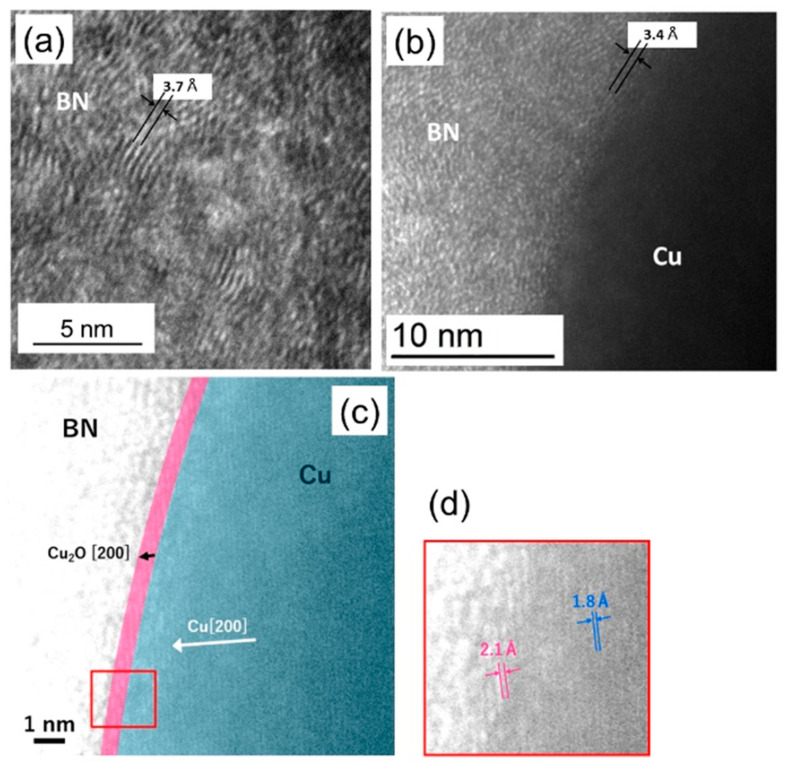
High resolution STEM images of BNNT near the Cu core. (**a**) BN region showing turbostratic structure and layer spacing; (**b**) interface region between BN and Cu. The layer spacing corresponds to that of h-BN; (**c**) interface between BN and Cu. At the interface, Cu_2_O is observed; (**d**) magnified and brighter image of Cu_2_O/Cu interface.

Figure 5 shows a high-resolution STEM image of the material prepared under the same condition as in Figure 4. The materials were scraped off the Si substrate onto the TEM grid. In Figure 5a, the contrast in the BN region shows an ~3.7 Å lattice spacing, which corresponds to BN (002). However, the ordering is not uniform, which can be assigned to turbostratic BN [32]. The sheets are curled and change their direction in a rather short range (~5 nm in Figure 5a). Better layer ordering with the spacing of 3.4 Å (bulk h-BN has 3.42 Å [1]) parallel to the Cu core surface is observed near the interface (Figure 5b). Figure 5c,d show the lattice spacing of 2.1 Å at the core surface for several layers, which is distinctly different from the inside of the Cu region (1.8 Å). This lattice spacing (2.1 Å) corresponds to that of Cu_2_O [33].

**Figure 6:**
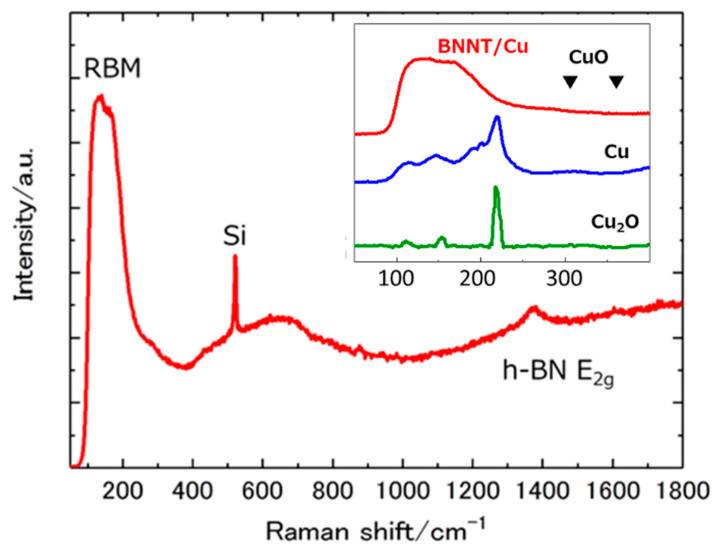
Raman spectrum of BNNT/Cu (web-like region of Figure 3). The inset compares low frequency region of BNNT/Cu (main panel), Cu, Cu_2_O [36], and CuO [37], two peaks are shown as triangles.

We examined the Raman spectrum of the sample by focusing the microscope to ~5 μm using a confocal aperture. Figure 6 shows the Raman spectrum from the web-like region in Figure 3. Along with the E_2g_ mode of h-BN at 1380 cm^-1^, broad structures between 100 and 250 cm^−1^ were observed. This region is characteristic of the radial breathing mode (RBM) of the BNNTs. It has been predicted that only single walled and double walled BNNTs show the RBM [9,34,35], and experimental observations cannot be found in the literature to the authors’ knowledge. The Cu catalyst obtained by reducing in H_2_ plasma was also measured using a Raman microscope. It is shown in the inset along with the RBM region of BNNT/Cu and Raman spectra of Cu_2_O and CuO from the literature. Based on the comparison, it is clearly shown that the broad peak of BNNT/Cu does not originate from the Raman signal of Cu, Cu_2_O [36], CuO [37], and their superposition. Since the RBM wavenumber varies with the diameter of the BNNT, the result is reasonably explained by assuming that the web-like structure consists of single or double walled BNNT with various diameters. The minimum diameter was estimated to be 2.3 nm from the higher wavenumber edge of the Raman low wavenumber structure and the relationship between the diameter and RBM frequency (d(nm) =246/ω(cm^−1^)) [38]. The maximum diameter cannot be evaluated because of the low wavenumber limit of the Raman spectrometer (c.a. 100 cm^−1^).

Below, we discuss the growth mechanism. It has been established that CNTs can be grown from transition metal catalytic nanoparticles such as the Fe-Co alloy [39,40]. These metal nanoparticles make a melt at the growth temperature and act as the catalyst for the decomposition of the carbon source molecules (such as CH_3_OH, CO, C_2_H_2_, etc.). At the same time, carbon is dissolved in the metal nanoparticles and precipitates to make the CNTs. With a high concentration of catalyst in the vapor phase, such as metallocene, the core-shell structure containing the catalyst metal core in the CNT [41,42]. However, the CNT in such core-shell structures have highly-ordered rolled layer structures. This is because the one-dimensional structure comes from the rolling of the graphene sheets.

**Figure 7:**
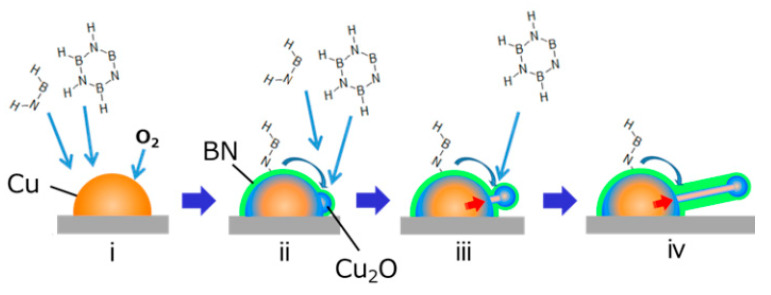
Growth model of turbostratic BN and Cu_2_O/Cu core-shell nanotubes.

In contrast, the BNNT grown in this experiment has three strange features. The first is the turbostratic nature with the randomly-oriented layered structures. The second is the Cu core wires densely contained in the BN. The third is the oxygen necessity for the growth of the BNNTs. A model to explain these features is illustrated in Figure 7. The first feature shows the one-dimensional growth did not originate from the rolling of the h-BN sheet. We consider the second and the third features to be related to the dynamic interaction between Cu nanoparticles and oxygen [43,44] with an enhanced migration [45]. Operando atomic resolution TEM observations of Cu nanoparticle catalysts in an oxygen atmosphere have recently been reported. In those studies, the dynamic shape change and motion of the Cu nanoparticles with dynamic reaction with oxygen (4Cu + O_2_ ⇄ 2Cu_2_O) were observed. The surface of the Cu region facing the turbostratic BN was Cu_2_O, which clearly shows that the BN growth was catalyzed by Cu_2_O. The change of the catalytic activity in CVD by the Cu surface oxidization to Cu_2_O was observed for the growth of graphene [46,47]. Based on these observations, we speculate that the growth mechanism is as follows: (i) Cu is oxidized to form Cu_2_O, (ii) Cu_2_O catalyzes the growth of turbostratic BN, (iii) the BN cover prevents oxygen intake and release from the Cu/Cu_2_O particle. If there is a hole in the BN, the Cu/Cu_2_O dynamic motion makes a protrusion of Cu, and (iv) a one-dimensional structure is formed by dynamic motion of the Cu and Cu_2_O near equilibrium. If the dynamic motion of Cu/Cu_2_O is fast, thin straight wires form (Figure 3); if it is slow, meandering structures form (Figure 4). This mechanism, the one-dimensional growth not coming from the rolling of the BN sheet, is the distinct difference from the CNT growth catalyzed by the transition metal nanoparticles.

## 4. Conclusions

We found that a core-shell nanotubular structure of turbostratic boron nitride and Cu is formed by plasma-assisted CVD using borazine, oxygen and an inert gas. Nanotubes were not formed without oxygen supply. Very thin (less than 1 nm) Cu_2_O was observed at the interface of the core Cu and shell BN, which probably works as the catalyst for the growth. A low frequency Raman signal was observed from the very thin web-like structures and was attributed to the radial breathing mode. It is difficult to consider that the growth mechanism is similar to that of the catalyst-filled core-shell carbon nanotubes. We point out the dynamic motion of the Cu/Cu_2_O system near equilibrium as the mechanism of one-dimensional growth. 

## Data Availability

Not applicable.
